# Draft Genome Sequence of *Aspergillus awamori* IFM 58123^NT^

**DOI:** 10.1128/MRA.01453-18

**Published:** 2019-01-24

**Authors:** Masaaki Shimizu, Yoko Kusuya, Yikelamu Alimu, Cai Bian, Hiroki Takahashi, Takashi Yaguchi

**Affiliations:** a Faculty of Science, Chiba University, Chiba, Japan; b Medical Mycology Research Center, Chiba University, Chiba, Japan; c Molecular Chirality Research Center, Chiba University, Chiba, Japan; Broad Institute of MIT and Harvard University

## Abstract

Species of the Aspergillus section *Nigri* are taxonomically very complex. The taxonomic assignment of Aspergillus awamori is unclear. Here, we present the draft genome sequence of A. awamori strain IFM 58123^NT^.

## Abstract (precis)

Species of the Aspergillus section *Nigri* are taxonomically very complex. The taxonomic assignment of Aspergillus awamori is unclear.

## ANNOUNCEMENT

Aspergillus awamori belongs to the Aspergillus section *Nigri*, which is composed of Aspergillus niger and related black aspergilli (1). These species are the second most common isolates from clinical specimens next to those of Aspergillus fumigatus and are also known as common food spoilage fungi (2). On the other hand, they are used for the production of enzymes and organic substances by fermentation (2, 3). Despite the vast diversity among these species, the morphological and molecular similarities prevent accurate classification among them (4). The taxonomy of Aspergillus section *Nigri* has been revised several times based on molecular phylogenetic methods. Most of the strains previously classified as A. awamori have been reclassified as Aspergillus welwitschiae (5). One cause of this misidentification problem is the poor resolution of molecular classification based on the limited number of genes. Thus, the whole-genome sequence of A. awamori strain IFM 58123^NT^ should help discern the phylogenetic complexity of the Aspergillus section *Nigri* species and develop industrial applications and accurate measures of food contamination.

A. awamori IFM 58123^NT^, stored at the Medical Mycology Research Center at Chiba University (IFM strains) in Japan, was used in this study. No living type material of A. awamori exists, and Al-Musallam (6) revised the taxonomy of black Aspergillus spp. using cluster analysis involving all available morphological and cultural parameters; as a result, strain NRRL 4948 (= CBS 557.65) was designated the neotype strain of A. awamori (6). The nucleic acid sequence containing the calmodulin gene of strain IFM 58123^NT^ was 100% (543/543 bp) identical to that of NRRL 4948 (= CBS 557.65, GenBank accession number KF288119). Therefore, the taxonomic position of IFM 58123^NT^ was confirmed. Following incubation in potato dextrose broth (PDB) at 37°C for 1 day, the genomic DNA of A. awamori IFM 58123^NT^ was extracted by phenol-chloroform extraction and using a NucleoBond AXG column (Macherey-Nagel, Düren, Germany) with NucleoBond buffer set III (Macherey-Nagel). The DNA libraries were prepared with the SMRTbell template prep kit 1.0 (Pacific Biosciences, Menlo Park, CA, USA), and genome sequencing was performed using a Pacific Biosciences RS II system. After filtering the reads by PreAssembler Filter version 1 (minimum subread length, 500 bp; minimum polymerase read quality, 0.80; minimum polymerase read length, 100 bp) in SMRT Analysis (version 2.3; Pacific Biosciences), we obtained a total of 622,132 reads summing 6,693,972,385 bp. The draft genome of A. awamori IFM 58123^NT^ was assembled with RS_HGAP_Assembly.3 protocol in SMRT Analysis (7) by Takara Bio (Mie, Japan). The draft genome contains 33 scaffolds, with a total size of 38,597,812 bp (49.35% G+C content). The *N*_50_ value and the maximum scaffold size were 4,298,649 bp and 6,450,824 bp, respectively. Prediction of protein-coding genes was performed using AUGUSTUS (version 2.5.5) with the option “–species=aspergillus_oryzae” (8), and a total of 11,224 genes were observed. The 310 tRNAs and 135 rRNAs were predicted by tRNAscan-SE (version 1.3.1) (9) and RNAmmer (version 1.2) (10), respectively.

### Data availability.

The whole-genome sequences of A. awamori IFM 58123^NT^ have been deposited at DDBJ/EMBL/GenBank under the accession numbers BDHI01000001 to BDHI01000033. The raw sequencing reads have been submitted to the SRA under the accession number DRA007470.
